# Raw data for the identification of SUMOylated proteins in *S. cerevisiae* subjected to two types of osmotic shock, using affinity purification coupled with mass spectrometry

**DOI:** 10.1016/j.dib.2014.11.003

**Published:** 2014-11-27

**Authors:** Tharan Srikumar, Megan C. Lewicki, Brian Raught

**Affiliations:** a Princess Margaret Cancer Centre, University Health Network, Canada; b Department of Medical Biophysics, University of Toronto, Toronto, ON, Canada

## Abstract

The small ubiquitin-related modifier (SUMO) “stress response” (SSR) is a poorly understood evolutionarily conserved phenomenon in which steady-state SUMO conjugate levels are dramatically increased in response to environmental stresses. Here we describe the data acquired using affinity-purification coupled with mass spectrometry to identify proteins that are SUMOylated in response to two different types of osmotic stress, 1 M sorbitol and 1 M KCl. The mass spectrometry dataset described here has been uploaded to the MassIVE repository with ID: MSV000078739, and consists of 32 raw MS files acquired in data-dependent mode on a Thermo Q-Exactive instrument. iProphet-processed MS/MS search results and associated SAINT scores are also included as a reference. These data are discussed and interpreted in “The *S. cerevisiae* SUMO stress response is a conjugation–deconjugation cycle that targets the transcription machinery”, by Lewicki et al. in the Journal of Proteomics, 2014 [1].

## Specifications table

**Table:** 

Subject area	Biology
More specific subject area	Proteomics, Protein–protein interactions
Type of data	Mass spectrometry RAW files
How data was acquired	Mass Spectrometry (Thermo Q-Exactive)
Data format	RAW unprocessed files
Experimental factors	*Saccharomyces cerevisiae* cultures stressed with 1 M sorbitol or 1 M KCl
Experimental features	AP-MS of SUMO conjugated proteins
Data source location	MassIVE
Data accessibility	Available on MassIVE, ID: MSV000078739.

### **Value of the data**

• Rigorously controlled dataset of SUMOylated proteins in *S. cerevisiae*, identified in untreated cultures, and following two different osmotic stressors (1 M sorbitol and 1 M KCl).
• ~200 SUMOylated proteins identified, with ~50 showing >2 fold-change following both types of osmotic stress.
• This work identifies a “core” group of proteins (primarily consisting of transcription factors and chromatin remodeling proteins) that are hyperSUMOylated in response to osmotic stress.

## 1 Data, experimental design, materials and methods

AP-MS was used to identify SUMO-conjugated proteins in unstressed (*i.e.* isosmotic conditions) and stressed (subjected to two different types of osmotic shock) budding yeast cultures. Semi-quantitative MS revealed a “core” group of proteins, consisting primarily of transcription factors and chromatin remodeling proteins, that display ≥2-fold increase in SUMOylation in response to osmotic shock.

### 1.1 SUMO conjugate affinity purification

The BY4741 *S. cerevisiae* strain was transformed with a plasmid coding for a galactose-inducible yeast HisFlag-SUMO protein (HF-SUMO), and putative SUMO conjugates were purified from 250 ml cultures grown overnight in Ura- media with 2% raffinose (supplemented with 0.2% galactose for induced samples) to an OD_600_ of 0.6. Induced and uninduced cells were treated with 1 M sorbitol (5 min) or 1 M KCl (15 min). Cells were collected by centrifugation at 4000*g* for 4 min at 4 °C, and washed once with 1 ml of 10% TCA before snap-freezing in an ethanol–dry ice bath. Frozen pellets were resuspended in lysis buffer (0.1 N NaOH, 2% SDS) and heated to 90 °C for 10 min. Lysates were sonicated for 10 s (at power setting 5) and cleared by centrifugation at 1500*g* for 5 min. Cleared lysates were diluted in 9 ml of PBS containing 250 U Turbonuclease (Biovision). HisFlag-SUMO conjugated proteins were affinity purified by incubating the diluted lysate with 150 µl of packed TALON beads (Clontech) at 4 °C for 1 h. Beads were collected and washed 4× with 1 ml of 50 mM ammonium bicarbonate (ammbic) pH 8.3. Washed beads were resuspended in 400 µl of 50 mM ammbic pH 8.3 containing 2 µg of LysC and 4 µg trypsin, and incubated for 16 h at 37 °C on an end-over-end rotator. The supernatant was collected and dried to completion in a Speedvac. Peptides were resuspended in 0.1% formic acid and subjected to LC-MS/MS analysis.

### 1.2 Mass spectrometry

Peptides were loaded onto a 75 µm ID, 50 cm long EasySPRAY column (Thermo Fisher Scientific) packed with 2 µm C18 reversed-phase material. Peptides were subjected to nanoflow liquid chromatography-electrospray ionization-tandem mass spectrometry (nLC-ESI-MS/MS) using a 100 min reversed phase (10–40% acetonitrile, 0.1% formic acid) buffer gradient running at 250 nL/min on a Proxeon EASY-nLC 1000 pump in-line with a Q-Exactive mass spectrometer (Thermo Fisher Scientific). Parent ion scans were performed at a resolving power of 70,000 with an AGC target of 3e6 across a scan range of 400–2000 *m/z* in profile mode. Up to the 15 most intense ions were selected for HCD with NCE of 27 and underfill of 1%. Chromatographic peak width and dynamic exclusion time was set to 15 s. Fragmentation spectra were acquired at a resolution of 17,500 with an AGC target of 2e5 in centroid mode.

### 1.3 Mass spectrometry data analysis

32 raw files were analyzed in the Prohits suite (ver. 3.0.1) against the yeast ORF translation (Refseq v57, appended with reverse sequence and cRAP protein sequences). The sample set consisted of 3 biological replicates for each condition (uninduced, untreated, 1 M sorbitol and 1 M KCl) with at least 2 replicate injections. Replicate injections were grouped for SAINT analysis (detailed in **File Summary**), and parameters set as follows: trypsin was set as the digestion enzyme with up to 2 missed cleavages permitted; acetylated protein N-termini, deamidation at glutamine and aspartate; ubiquitylation of lysines and oxidized methionines were set as variable modifications. A 10 ppm window was used for the parent and 0.02 Da for fragment ions. Two search algorithms were used: X!Tandem (ver. Cyclone TPP) [2] and Comet (ver. 2013.02rev0) [3]. Results from both search engines were combined using iProphet (ver. 4.6rev3) [4]. Protein identifications made at a 1% FDR (~0.75 iProphet score) were used for further analysis. SAINT analysis [5] was used to identify proteins found specifically under Gal-induced conditions, using the uninduced samples (*i.e.* cells grown in raffinose alone) as controls. Proteins with a SAINT score ≥0.9 in the unstressed (Gal induced) or stressed conditions (1 M sorbitol or 1 M KCl) were deemed SUMOylated proteins. 1 spectral count was added to the total for each of these proteins (to avoid division by 0), and those that displayed ≥2 fold-change in spectral counts *vs*. untreated in both stressed conditions (1 M sorbitol and 1 M KCl) were categorized as the “core” group of stress responsive SUMO targets [1].
